# Advanced glycation end products evoke endothelial cell damage by stimulating soluble dipeptidyl peptidase-4 production and its interaction with mannose 6-phosphate/insulin-like growth factor II receptor

**DOI:** 10.1186/1475-2840-12-125

**Published:** 2013-08-28

**Authors:** Yuji Ishibashi, Takanori Matsui, Sayaka Maeda, Yuichiro Higashimoto, Sho-ichi Yamagishi

**Affiliations:** 1 Department of Pathophysiology and Therapeutics of Diabetic Vascular Complications, Kurume University School of Medicine, 67 Asahi-machi, Kurume, 830-0011, Japan; 2 Department of Medical Biochemistry, Kurume University School of Medicine, Kurume, 830-0011, Japan

**Keywords:** AGEs, RAGE, DPP-4, Mannose 6-phosphate/IGF-II receptor

## Abstract

**Background:**

Advanced glycation end products (AGEs) and receptor RAGE interaction play a role in diabetic vascular complications. Inhibition of dipeptidyl peptidase-4 (DPP-4) is a potential therapeutic target for type 2 diabetes. However, the role of DPP-4 in AGE-induced endothelial cell (EC) damage remains unclear.

**Methods:**

In this study, we investigated the effects of DPP-4 on reactive oxygen species (ROS) generation and RAGE gene expression in ECs. We further examined whether an inhibitor of DPP-4, linagliptin inhibited AGE-induced soluble DPP-4 production, ROS generation, RAGE, intercellular adhesion molecule-1 (ICAM-1) and plasminogen activator inhibitor-1 (PAI-1) gene expression in ECs.

**Results:**

DPP-4 dose-dependently increased ROS generation and RAGE gene expression in ECs, which were prevented by linagliptin. Mannose 6-phosphate (M6P) and antibodies (Ab) raised against M6P/insulin-like growth factor II receptor (M6P/IGF-IIR) completely blocked the ROS generation in DPP-4-exposed ECs, whereas surface plasmon resonance revealed that DPP-4 bound to M6P/IGF-IIR at the dissociation constant of 3.59 x 10^-5^ M. AGEs or hydrogen peroxide increased soluble DPP-4 production by ECs, which was prevented by *N*-acetylcysteine, RAGE-Ab or linagliptin. Linagliptin significantly inhibited the AGE-induced ROS generation, RAGE, ICAM-1 and PAI-1 gene expression in ECs.

**Conclusions:**

The present study suggests that AGE-RAGE-induced ROS generation stimulates the release of DPP-4 from ECs, which could in turn act on ECs directly via the interaction with M6P/IGF-IIR, further potentiating the deleterious effects of AGEs. The blockade by linagliptin of positive feedback loop between AGE-RAGE axis and DPP-4 might be a novel therapeutic target for vascular injury in diabetes.

## Background

The pathological role of the non-enzymatic modification of amino groups of proteins, nucleic acids and lipids by reducing sugars such as glucose, a process that is also known as “Maillard reaction”, has become increasingly evident in various types of diseases [1-3]. It is now well established that early glycation products undergo further progressive modification over time *in vivo* to the formation of irreversibly cross-linked senescent macroprotein derivatives termed “advanced glycation end products (AGEs)” [1-3]. The formation and accumulation of AGEs in various tissues have been known to progress at a physiological aging and at an accelerated rate under hyperglycemic conditions [1-3]. There is accumulating evidence that AGEs elicit oxidative stress generation and subsequently evoke inflammatory and thrombogenic reactions in a variety of cells through the interaction with the receptor for AGEs (RAGE), thereby being involved in vascular complications in diabetes [4-9].

Dipeptidyl peptidase-4 (DPP-4), also known as CD26, is a type II transmembrane glycoprotein expressed on various cell types with multifunctional properties [10,11]. DPP-4 not only plays a role in T cell activation and proliferation, but also modulates the physiological activity of many regulatory peptides, because it is involved in the cleavage of N-terminal amino acids from several chemokines and neuropeptides [10,11]. Incretins such as glucagon-like peptide-1 (GLP-1) and glucose-dependent insulinotropic polypeptides (GIP) are gut hormones secreted from L and K cells in the intestine in response to food intake, respectively [12,13], both of which are target proteins of DPP-4 and rapidly degraded and inactivated by this proteolytic enzyme [10,11]. Since GLP-1 and GIP augment glucose-induced insulin release from pancreatic b-cells, suppresses glucagon secretion, and slows gastric emptying [12,13], inhibition of DPP-4 has been proposed as a potential therapeutic target for the treatment of type 2 diabetes. However, it remains unclear DPP-4 inhibition could have beneficial effects on AGE-exposed endothelial cells (ECs). In other words, whether DPP-4 itself is involved in vascular injury in diabetes remains unknown. DPP-4 and D-Mannose-6-phosphate/insulin-like growth factor II receptor (M6P/IGF-IIR) interaction contributes to T cell activation [14]. Therefore, in this study, we first investigated whether DPP-4 could directly act on human umbilical vein ECs (HUVECs) to stimulate reactive oxygen species (ROS) generation and RAGE gene induction via the interaction with M6P/IGF-IIR. We next examined the effects of AGEs on soluble DPP-4 production released from HUVECs. We further studied whether an inhibitor of DPP-4, linagliptin inhibited the AGE-induced soluble DPP-4 production, ROS generation, RAGE, intercellular adhesion molecule-1 (ICAM-1) and plasminogen activator inhibitor-1 (PAI-1) gene expression in HUVECs.

## Methods

### Materials

An inhibitor of DPP-4, linagliptin was generously gifted from Boehringer Ingelheim (Ingelheim, Germany). Bovine serum albumin (BSA) (essentially fatty acid free and essentially globulin free, lyophilized powder), D-Mannose-6-phosphate (M6P) and *N*-acetylcysteine (NAC) were purchased from Sigma (St. Louis, MO, USA). D-glyceraldehyde from Nakalai Tesque (Kyoto, Japan). Recombinant human DPP-4 from R&D systems (Minneapolis, MN, USA). Hydrogen peroxide (H_2_O_2_) from Wako Pure Chemical Industries Ltd. (Osaka, Japan). Antibody (Ab) directed against human M6P/insulin-like growth factor II receptor (IGF-IIR) (M6P/IGF-IIR-Ab) and DPP-4 from Santa Cruz Biotechnology Inc. (Delaware, CA, USA).

### Cells

HUVECs were cultured in endothelial basal medium supplemented with 2% fetal bovine serum, 0.4% bovine brain extracts, 10 ng/ml human epidermal growth factor and 1 μg/ml hydrocortisone according to the supplier’s instructions (Clonetics Corp., San Diego, CA). DPP-4 or AGE treatment was carried out in a medium lacking epidermal growth factor and hydrocortisone.

### Dihydroethidium (DHE) staining

HUVEC were treated with or without the indicated concentrations of DPP-4, 100 μg/ml AGE-BSA or 100 μg/ml non-glycated BSA in the presence or absence of 50 μM M6P, 5 μg/ml M6P/IGF-IIR-Ab, 10 nM or 0.5 μM linagliptin for 4 hr, and then the cells were incubated with phenol red free Dulbecco's Modified Eagle Medium containing 3 μM DHE (Molecular Probes Inc., Eugene, OR, USA). After 15 minutes, the cells were imaged under a laser-scanning confocal microscope. Superoxide generation was evaluated by intensity of DHE staining. The intensity was analyzed by microcomputer-assisted NIH image.

### Surface plasmon resonance (SPR)

Recombinant human IGF-IIR (100 μg/ml, R&D system) was immobilized via the amino groups to CM5 sensor chip (GE Healthcare, Buckinghamshire, UK) with the aid of 1-ethyl-3-(3-dimethylaminopropyl)-carbodiimide and *N*-hydroxysuccinimide. For affinity measurements, the association and dissociation phases were monitored in a BIAcore 1000 (GE Healthcare). Recombinant human DPP-4 was injected into the flow cell at concentrations of 0.1 and 0.3 μM at a flow rate of 10 μl/min at 25°C. The sensor chip was regenerated with pulses of 20 mM Tris–HCl buffer (pH 8.0) containing 6 M urea to the baseline level, followed by an extensive washing with the running buffer. Control experiments were performed with IGF-IIR-free channel on the same sensor chip. From the assay curves obtained, the control signals, reflecting the bulk effect of buffer, were subtracted using BIA-evaluation 4.1 software (GE Healthcare). Equilibrium dissociation constant (*K*_D_) was determined using the equation for 1:1 Langmuir binding.

### Real-time reverse transcription-polymerase chain reactions (RT-PCR)

HUVEC were treated with or without the indicated concentrations of DPP-4, 100 μg/ml AGE-BSA or 100 μg/ml non-glycated BSA in the presence or absence of 10 nM or 0.5 μM linagliptin for 4 hr. Then total RNA was extracted with RNAqueous-4PCR kit (Ambion Inc., Austin, TX, USA) according to the manufacturer’s instructions. Quantitative real-time RT-PCR was performed using Assay-on-Demand and TaqMan 5 fluorogenic nuclease chemistry (Applied Biosystems, Foster city, CA, USA) according to the supplier’s recommendation. IDs of primers for human RAGE, ICAM-1, PAI-1, β-actin and 18S gene were Hs00153957_m1, Hs00164932_m1, Hs01126606_m1, Hs99999903_m1, and Hs99999901_s1, respectively.

### Preparation of AGE-BSA

AGE-BSA was prepared as described previously [15]. In brief, BSA (25 mg/ml) was incubated under sterile conditions with 0.1 M glyceraldehyde in 0.2 M NaPO_4_ buffer (pH 7.4) for 7 days. Then unincorporated sugars were removed by PD-10 column chromatography and dialysis against phosphate-buffered saline. Control non-glycated BSA was incubated in the same conditions except for the absence of reducing sugars. Preparations were tested for endotoxin using Endospecy ES-20S system (Seikagaku Co., Tokyo, Japan); no endotoxin was detectable.

### Preparation of Ab raised against RAGE (RAGE-Ab)

Ab directed against human RAGE was prepared as described previously [16].

### Soluble DPP-4 production

HUVECs were treated with or without 100 μg/ml AGE-BSA, 100 μg/ml non-glycated BSA or the indicated concentrations of H_2_O_2_ in the presence or absence of 1 mM NAC, 5 μg/ml RAGE-Ab or 10 nM linagliptin for 24 hr. Conditioned medium were collected and concentrated 20-fold using an Amicon ultrafiltration system (5000-kDa cutoff, Merck Millipore, Darmstadt, Germany) according to the method described previously [16]. Then 20 μg proteins were separated by SDS-PAGE and transferred to polyvinylidene difluoride membranes as described previously [17]. Membranes were probed with Ab directed against human DPP-4, and then immune complexes were visualized with an enhanced chemiluminescence detection system (Amersham Bioscience, Buckinghamshire, United Kingdom).

### Statistical analysis

Unless otherwise indicated, all values were presented as means ± SE from at least 3 independent experiments. Statistical analyses were performed by one-way ANOVA followed by the Scheffe F test for multiple comparisons, and p < 0.05 was considered statistically significant. All statistical analyses were performed with the use of the PASW Statistics system (version 18.0; IBM Corporation, New York, NY, USA).

## Results

**Figure 1:**
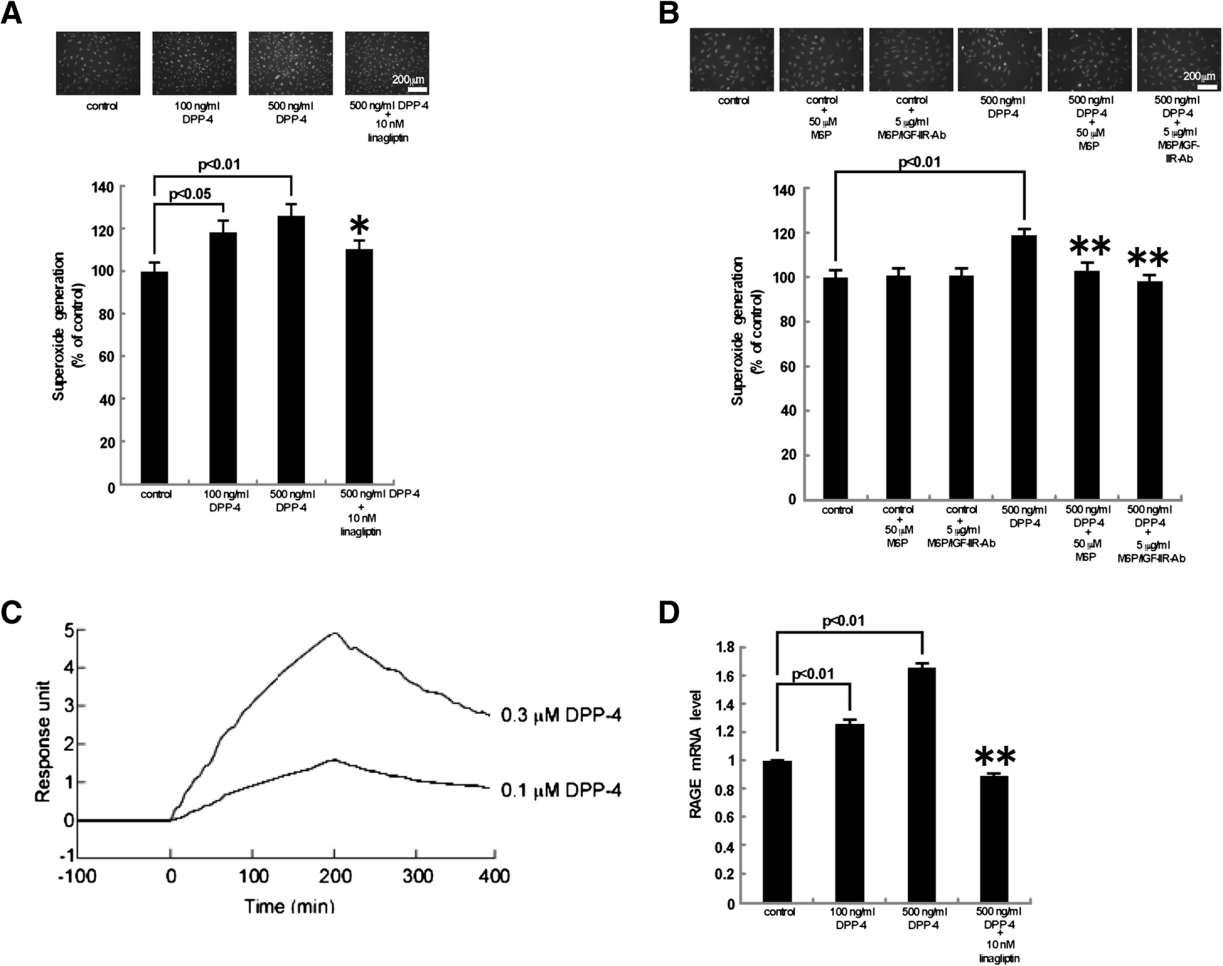
**Effects of DPP-4 on ROS generation (A and B) and RAGE gene expression (D) in HUVECs and (C) representative binding sensorgram of DPP-4 to immobilized M6P/IGF-IIR.** HUVEC were treated with or without the indicated concentrations of DPP-4 in the presence or absence of 50 μM M6P, 5 μg/ml M6P/IGF-IIR-Ab, or 10 nM linagliptin for 4 hr. **(A and B)** Then the cells were incubated with DHE. Upper panel shows typical microphotographs of the cells. Lower panel shows quantitative data of ROS generation evaluated by fluorescent intensity. **(A)***N* = 12 per group. **(B)***N* = 27 per group. **(D)** Total RNAs were transcribed and amplified by real-time PCR. Data were normalized by the intensity of β-actin mRNA-derived signals and then related to the value obtained with control. *N* = 3 per group. **(C)** DPP-4 at 0.1 and 0.3 μM was injected on the sensor chip immobilized M6P/IGF-IIR. *N* = 3 per group. * and **, p < 0.05 and p < 0.01 compared to the value with control, respectively.

We first examined the effects of DPP-4 on ROS generation in HUVECs. As shown in Figure 1A and 1B, DPP-4 dose-dependently increased superoxide generation in HUVECs; 500 ng/ml DPP-4-induced increase in ROS generation was completely blocked by the treatment with 10 nM linagliptin, 50 μM M6P or 5 μg/ml M6P/IGF-IIR-Ab. M6P or M6P/IGF-IIR alone did not affect superoxide generation in HUVECs. Figure 1C shows the representative binding sensorgram of 0.1 and 0.3 μM DPP-4 to immobilized M6P/IGF-IIR. SPR analysis revealed that DPP-4 bound to M6P/IGF-IIR; *K*_D_ value was 3.59 × 10^-5^ ± 1.35 × 10^-5^ M. Furthermore, DPP-4 dose-dependently RAGE gene expression in HUVECs, which was also blocked by linagliptin (Figure 1D).

**Figure 2:**
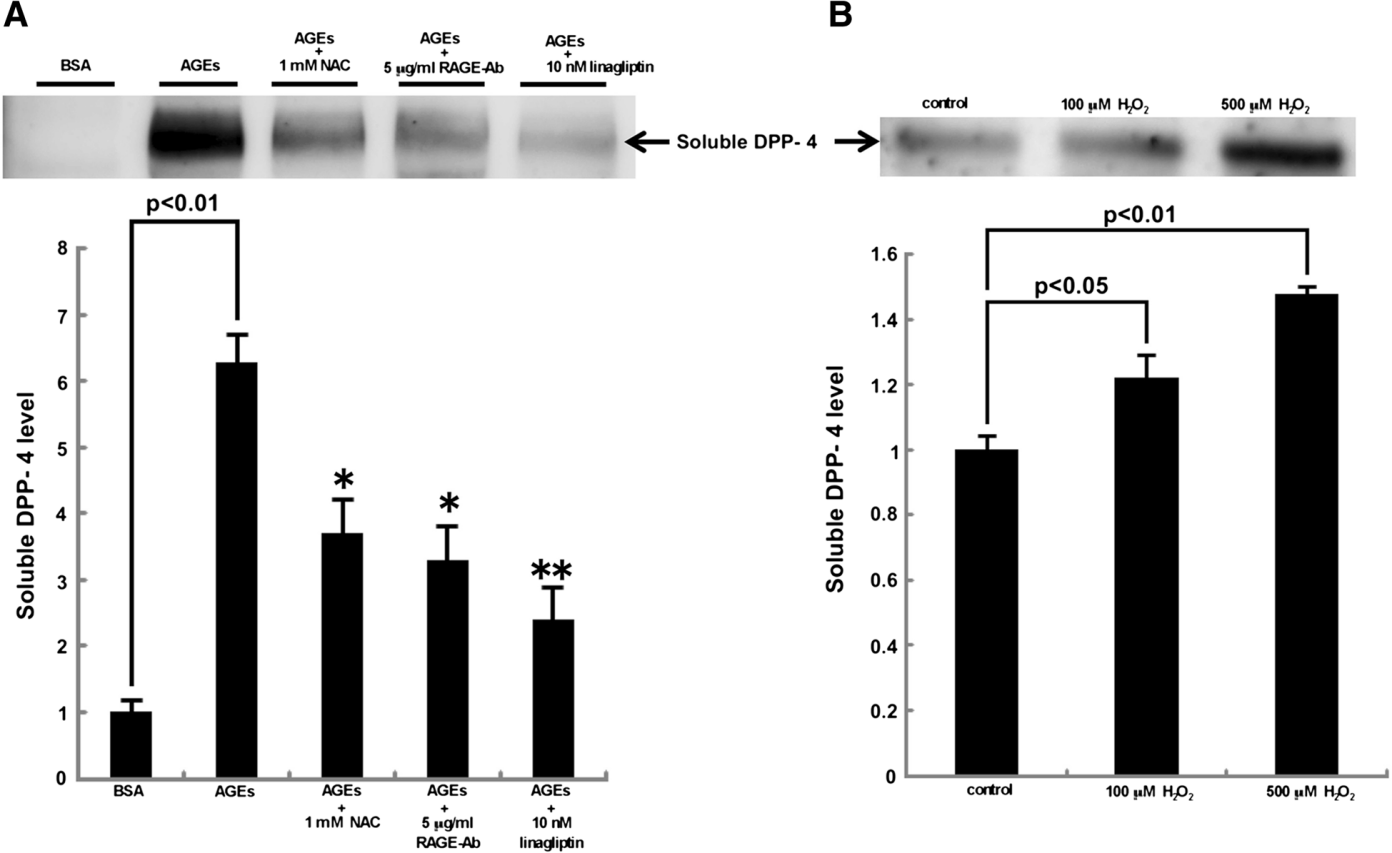
**Effects of AGEs on DPP-4 production released from HUVECs.** HUVECs were treated with or without 100 μg/ml AGE-BSA, 100 μg/ml non-glycated BSA or the indicated concentrations of H_2_O_2_ in the presence or absence of 1 mM NAC, 5 μg/ml RAGE-Ab or 10 nM linagliptin for 24 hr. Conditioned medium were collected and concentrated 20-fold using an Amicon ultrafiltration system. Then 20 μg proteins were separated by SDS-PAGE and transferred to polyvinylidene difluoride membranes. Soluble DPP-4 expression released from the cells was measured. Each upper panel shows the representative bands. Lower panel shows the quantitative data. **(A)***N* = 3 per group. **(B)***N* = 9 per group.

We next examined whether AGEs could stimulate soluble DPP-4 generation by HUVECs. As shown in Figure 2A, AGEs increased DPP-4 production released from HUVECs, which was significantly prevented by the treatment with an anti-oxidant, NAC, RAGE-Ab or linagliptin. Moreover, H_2_O_2_ dose-dependently stimulated the release of DPP-4 from HUVECs (Figure 2B).

**Figure 3:**
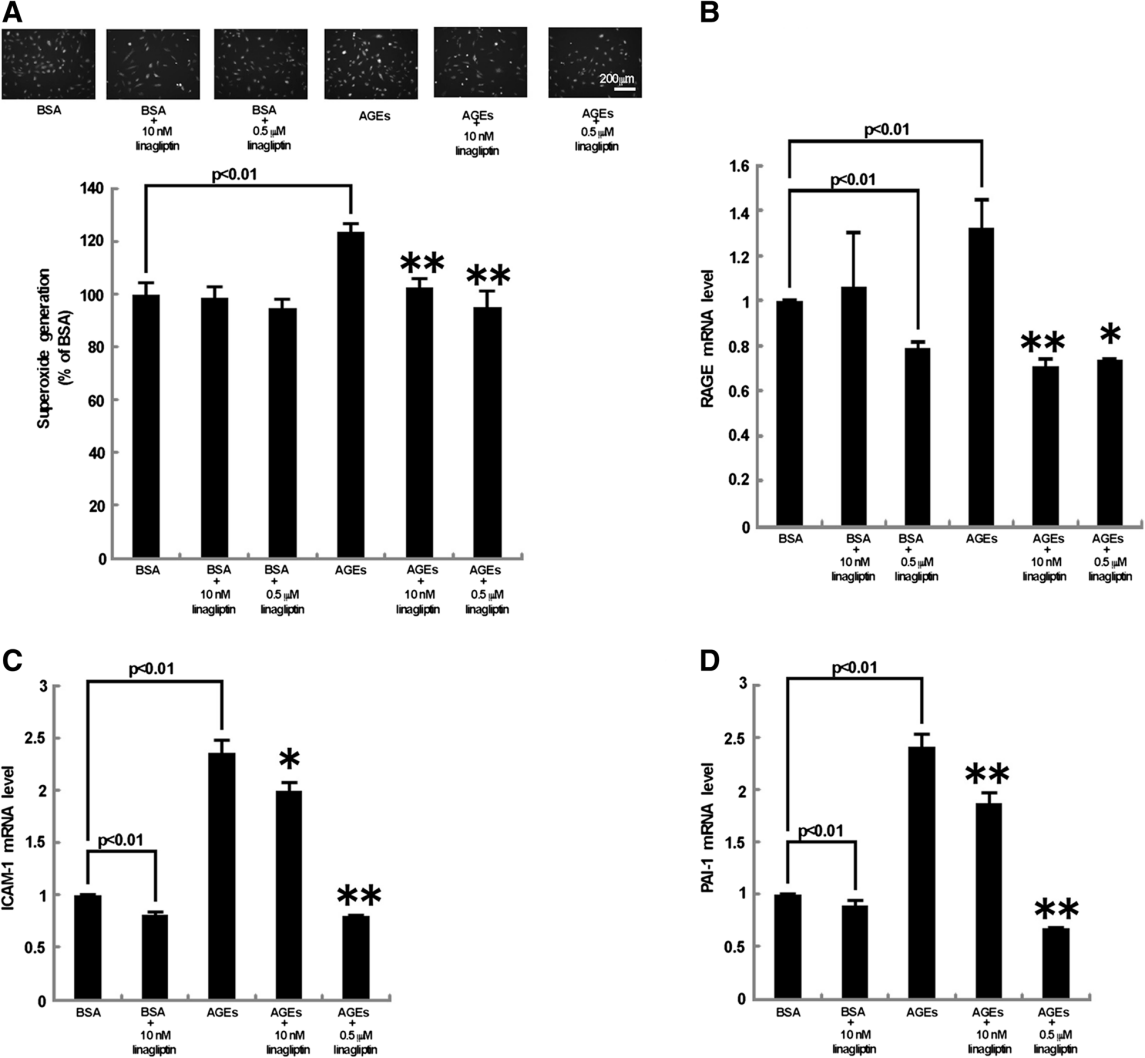
**Effects of linagliptin on AGE-exposed HUVECs.** HUVECs were treated with 100 μg/ml AGE-BSA or non-glycated BSA in the presence or absence of 10 nM or 0.5 μM linagliptin for 4 hr. **(A)** Cells were incubated with DHE. Upper panel shows typical microphotographs of the cells. Lower panel shows quantitative data of ROS generation evaluated by fluorescent intensity. *N* = 50 per group. **(B-D)** Total RNAs were transcribed and amplified by real-time PCR. Data were normalized by the intensity of β-actin **(B)** or 18S mRNA-derived signals **(C and D)** and then related to the value obtained with non-glycated BSA. **(B)***N* = 15 per group. **(B)***N* = 15 per group. **(C and D)***N* = 9 per group. * and **, p < 0.05 and p < 0.01 compared to the value with AGEs alone, respectively.

We further investigated the effects of linagliptin on AGE-exposed HUVEC. As shown in Figure 3, AGEs stimulated superoxide generation and up-regulated m RNA levels of RAGE, ICAM-1 and PAI-1 in HUVECs, all of which were significantly blocked by linagliptin.

## Discussion

### Role of DPP-4 in vascular injury

M6P/IGF-IIR has been shown to work as a receptor for DPP-4 in HUVECs and mediates its biological action, resultantly promoting transendothelial T cell migration, and an effect requires the enzymatic activity of DPP-4 [18]. In this study, we found for the first time that blocking the interaction of DPP-4 with M6P/IGF-IIR by the addition of excess amount of free M6P or M6P/IGF-IIR-Ab completely inhibited the DPP-4-induced increase in superoxide generation in HUVECs. Further, SPR analysis revealed that DPP-4 actually bound to M6P/IGF-IIR, and an inhibitor of DPP-4, linagliptin completely prevented the ROS generation and up-regulation of RAGE mRNA levels in DPP-4-exposed HUVECs. Since we have previously shown that AGEs stimulate RAGE gene expression in ECs via ROS generation [15], our present observations suggest that DPP-4 could increase ROS generation and subsequently RAGE gene overexpression in HUVECs through the interaction with M6P/IGF-IIR, whose ability is totally dependent on its intrinsic DPP-4 enzymatic activity. AGEs have been shown to impair the migration, adhesion and secretion potentials of late endothelial progenitor cells [19,20]. Further, AGE-RAGE interaction causes inflammation and thrombogenesis in diabetic vessels [21,22]. Therefore, given the atherosclerosis-promoting properties of AGE-RAGE axis, although the reason why DPP-4 caused vascular damage in diabetes remains unclear, DPP-4 itself might be involved in diabetic vascular injury. DPP-4 has been reported to not only stimulate proliferation of human coronary artery smooth muscle cells [23], but also contribute to monocyte migration, macrophage-mediated inflammatory reactions and tissue remodeling [24,25], thus supporting the speculation that DPP-4 itself might work as a risk factor for atherosclerosis.

### Crosstalk between AGE-RAGE axis and DPP-4

In this study, AGEs stimulated the release of DPP-4 from HUVECs, which was significantly inhibited by the treatment with an anti-oxidant, NAC, RAGE-Ab, or linagliptin. Moreover, H_2_O_2_ dose-dependently increased the production of soluble DPP-4 by HUVECs. So, the AGE-RAGE-induced ROS generation could be involved in soluble DPP-4 generation by HUVECs. The present findings have extended our previous observations [17] showing that serum levels of AGEs were independently correlated with circulating DPP-4 values in 432 consecutive outpatients and that AGEs significantly increase soluble DPP-4 release from cultured proximal tubular cells, one of the major cell types that expressed DPP-4 in humans [26]. Since we previously reported that AGEs at 100 μg/ml for 4 hr did not affect DPP-4 mRNA levels in HUVECs [27], the AGE-RAGE interaction might promote the proteolytic cleavage of membrane-bound DPP-4 from HUVECs via superoxide generation. Serum levels of AGEs are positively *rather than* inversely associated with soluble form of RAGE (sRAGE) (endogenous secretory RAGE plus cleaved RAGE) in both diabetic and non-diabetic subjects [28,29]. Therefore, although exogenously administered sRAGE was shown to block the harmful effects of AGEs in animals by acting as a decoy receptor, it is questionable that sRAGE in humans could also exert the same biological effect, because its serum concentration is 1000 times lower than needed for efficiently capturing and eliminating the circulating AGEs [30]. Moreover, engagement of RAGE with its ligand has been shown to promote the RAGE shedding [30,31]. These findings suggest that sRAGE level could reflect tissue RAGE expression and that AGEs might enhance the cleavage of DPP-4 from the cell membrane. Given the facts that serum DPP-4 activity is largely associated with circulating DPP-4 levels [10,32] and that 20% of incretins derived from gastrointestinal tract are still alive in the blood pool [33,34], cumulative hyperglycemia and resultant AGE accumulation might impair the incretins’ effects via elevation of circulating DPP-4 levels, further deteriorating glycemic control and thereby forming a vicious cycle in diabetic subjects. This scenario could support the clinical relevance of blockade of the pathological crosstalk between AGE-RAGE axis and DPP-4 by linagliptin in the treatment with type 2 diabetes.

### Protective role of linagliptin against AGE-RAGE-induced vascular damage in diabetes

In the present study, we found that linagliptin significantly inhibited the AGE-induced ROS generation, RAGE, ICAM-1 and PAI-1 gene expression in HUVECs. AGEs are reported to up-regulate RAGE gene expression in a variety of cells via ROS generation and induce activation of redox-sensitive transcriptional factor, NF-kB and subsequent ICAM-1 and PAI-1 gene induction [7,15,31,35-37]. Therefore, the AGE-RAGE-induced oxidative stress generation could further potentiate the harmful effects of AGEs via RAGE overexpression. So, linagliptin might inhibit the AGE-evoked inflammatory and thrombogenic responses in HUVECs by blocking the positive feedback loops between ROS generation and RAGE gene up-regulation. In this study, we could not clarify the mechanism by which linagliptin inhibited the AGE-induced ROS generation in HUVECs. However, we have previously shown that GLP-1 and GIP protect against AGE-induced HUVEC damage via anti-oxidative properties through the elevation of cyclic AMP, whose effect is augmented by the addition of DPP-4 inhibitor [27,38,39]. Since AGE-RAGE axis evokes ROS generation in ECs via NADPH oxidase activity, which is blocked by cAMP-elevating agents [6,7,15,40], linagliptin could enhance the beneficial effects of incretins on AGE-exposed HUVECs by inhibiting NADPH oxidase activity. Furthermore, we have recently found that linagliptin contains xanthine scaffold structure, which could inhibit xanthine oxidase activity *in vitro*[41]. The anti-oxidative unique properties of this drug might also be involved in the blockade of vicious cycle between ROS generation and RAGE gene induction. It is unlikely that linagliptin directly inhibited the AGE-RAGE interaction because highly sensitive 27-MHz quartz crystal microbalance analysis (Affinix Q; Initium, Tokyo, Japan) revealed that linagliptin can not bind to AGEs *in vitro* (data not shown).

One early phase of atherosclerosis involves the recruitment and firm adhesion of inflammatory cells to ECs, whose process is mediated by adhesion molecules such as ICAM-1 [42,43]. Further, attenuated fibrinolytic activity due to increased PAI-1 levels is prevalent in diabetic patients, thus contributing to the increased risk of atherothrombosis in these subjects [37,44,45]. Linagliptin may be a promising strategy for not only ameliorating hyperglycemia in type 2 diabetic patients, but also protecting against vascular injury by suppressing ICAM-1 and PAI-1 expression through blockade of the deleterious effects of AGE-RAGE axis partly via inhibition of DPP-4 and M6P/IGF-IIR interaction. In pre-specified meta-analysis of cardiovascular events in linagliptin or comparator-treated patients with type 2 diabetes mellitus, the hazard ratio for a composite of cardiovascular death, stroke, myocardial infarction, and hospitalization for unstable angina showed significantly lower risk with linagliptin than comparator [46]. Moreover, we have very recently found that DPP-4 inhibitor alogliptin treatment blocks the AGE-RAGE axis and resultantly reduces albuminuria in type 2 diabetes patients [47]. Fluorescent AGE levels have also been shown to be an independent marker of post-infarction heart failure development risk [48]. These data reinforce the important clinical implications of the present findings of linagliptin.

The peak plasma concentration of linagliptin after administration of single oral dose of 5 mg is reported to be about 10 nM [49]. So, the concentration of linagliptin having beneficial effects on HUVECs used in the present experiments (10 nM) may also be comparable to the therapeutic level which is achieved in the treatment for patients with type 2 diabetes.

### Limitations

Our study has several limitations that should be noted. First, we did not examine here the effect of M6P/IGF-IIR-Ab on the increase in ROS generation induced by AGEs or the increase in RAGE gene expression induced by DPP-4 and AGEs. Second, although mRNA levels of DPP-4 were not changed by the treatment with AGEs, the effect of linagliptin on membrane DPP-4 expression in AGE-exposed HUVECs remains unknown. Additional experiments would strengthen the present findings.

## Conclusions

**Figure 4:**
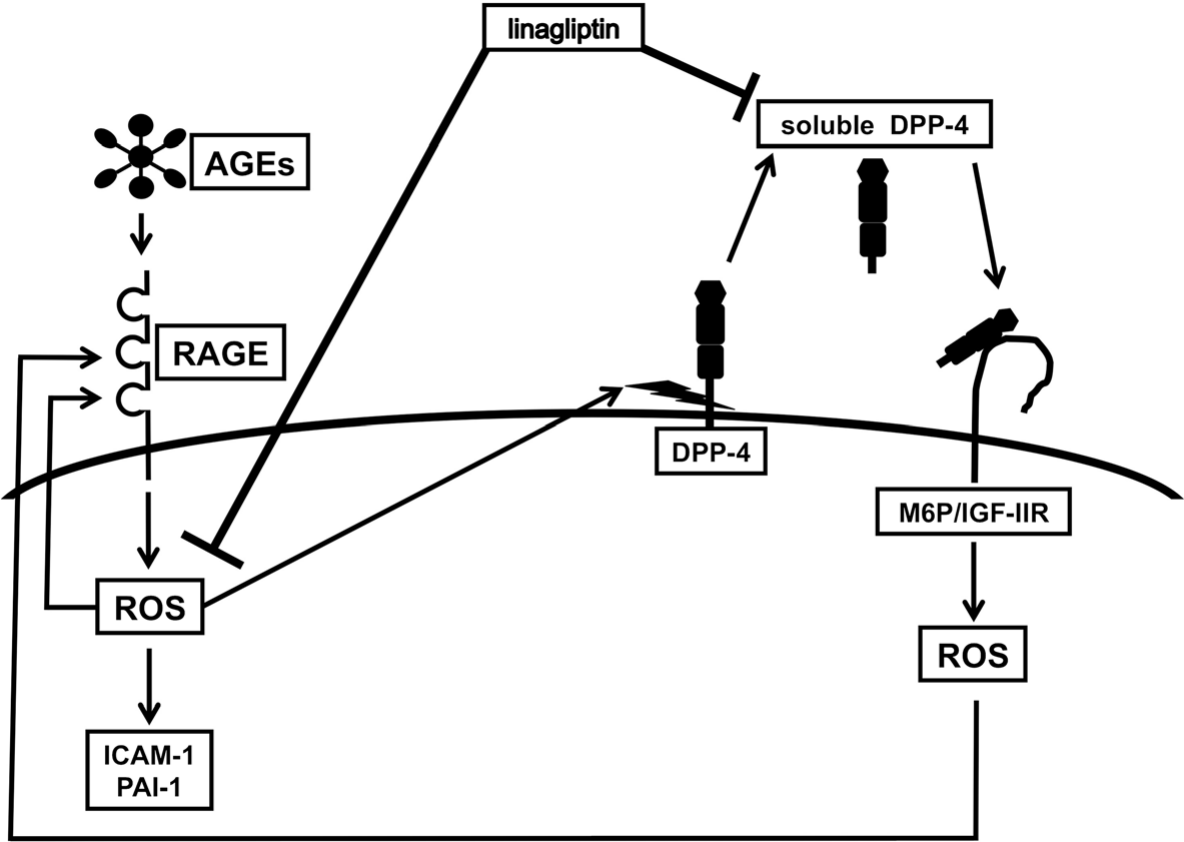
Possible crosstalk between AGE-RAGE axis and soluble DPP-4.

Our present observations suggest AGEs could stimulate the release of DPP-4 from HUVECs via RAGE-mediated ROS generation, which may further augment the AGE-RAGE signaling to EC damage through the interaction with M6P/IGF-IIR (Figure 4).

## Abbreviations

AGEs: Advanced glycation end products; RAGE: Receptor for AGEs; DPP-4: Dipeptidyl peptidase-4; GLP-1: Glucagon-like peptide-1; GIP: Glucose-dependent insulinotropic polypeptides; ECs: Endothelial cells; M6P/IGF-IIR: D-Mannose-6-phosphate/insulin-like growth factor II receptor; HUVECs: Human umbilical vein ECs; ROS: Reactive oxygen species; ICAM-1: Intercellular adhesion molecule-1; PAI-1: Plasminogen activator inhibitor-1; BSA: Bovine serum albumin; M6P: D-Mannose-6-phosphate; NAC: *N*-acetylcysteine; Ab: Antibody; IGF-IIR: Insulin-like growth factor II receptor; M6P/IGF-IIR-Ab: Ab raised against M6P/IGF-IIR; SPR: Surface plasmon resonance; KD: Dissociation constant; RT-PCR: Reverse transcription-polymerase chain reaction; RAGE-Ab: Ab raised against RAGE; sRAGE: Soluble form of RAGE.

## Competing interests

Dr. Yamagishi has received honoraria such as lecture fees from Boehringer Ingelheim and Eli Lilly. The authors declare that they have no competing interests.

## Authors’ contributions

YI, TM, SM, and YH acquired and interpreted data. SY mainly contributed to the present study, conceptualized and designed the study, acquired, analyzed, and interpreted data, and drafted the manuscript, and took responsibility for the integrity of the data and the accuracy of the data analysis. All authors read and approved the final manuscript.

## References

[B1] Vlassara H Bucala R Recent progress in advanced glycation and diabetic vascular disease: role of advanced glycation end product receptors Diabetes 1996 45 Suppl 3 S65 S66 8674896 10.2337/diab.45.3.s65

[B2] Brownlee M Cerami A Vlassara H Advanced glycosylation end products in tissue and the biochemical basis of diabetic complications N Engl J Med 1988 318 1315 1321 10.1056/NEJM198805193182007 3283558

[B3] Rahbar S Novel inhibitors of glycation and AGE formation Cell Biochem Biophys 2007 48 147 157 10.1007/s12013-007-0021-x 17709884

[B4] Yamamoto Y Kato I Doi T Yonekura H Ohashi S Takeuchi M Watanabe T Yamagishi S Sakurai S Takasawa S Development and prevention of advanced diabetic nephropathy in RAGE-overexpressing mice J Clin Invest 2001 108 261 268 11457879 10.1172/JCI11771 PMC203021

[B5] Wendt TM Tanji N Guo J Kislinger TR Qu W Lu Y Bucciarelli LG Rong LL Moser B Markowitz GS RAGE drives the development of glomerulosclerosis and implicates podocyte activation in the pathogenesis of diabetic nephropathy Am J Pathol 2003 162 1123 1137 10.1016/S0002-9440(10)63909-0 12651605 PMC1851245

[B6] Yamagishi S Imaizumi T Diabetic vascular complications: pathophysiology, biochemical basis and potential therapeutic strategy Curr Pharm Des 2005 11 2279 2299 10.2174/1381612054367300 16022668

[B7] Yamagishi S Nakamura K Matsui T Noda Y Imaizumi T Receptor for advanced glycation end products (RAGE): a novel therapeutic target for diabetic vascular complication Curr Pharm Des 2008 14 487 495 10.2174/138161208783597416 18289075

[B8] Jandeleit-Dahm K Cooper ME The role of AGEs in cardiovascular disease Curr Pharm Des 2008 14 979 986 10.2174/138161208784139684 18473849

[B9] Tahara N Yamagishi S Takeuchi M Honda A Tahara A Nitta Y Kodama N Mizoguchi M Kaida H Ishibashi M Positive association between serum level of glyceraldehyde-derived advanced glycation end products and vascular inflammation evaluated by [(18)F]fluorodeoxyglucose positron emission tomography Diabetes Care 2012 35 2618 2625 10.2337/dc12-0087 22912424 PMC3507595

[B10] Cordero OJ Salgado FJ Nogueira M On the origin of serum CD26 and its altered concentration in cancer patients Cancer Immunol Immunother 2009 58 1723 1747 10.1007/s00262-009-0728-1 19557413 PMC11031058

[B11] Yazbeck R Howarth GS Abbott CA Dipeptidyl peptidase inhibitors, an emerging drug class for inflammatory disease? Trends Pharmacol Sci 2009 30 600 607 10.1016/j.tips.2009.08.003 19837468

[B12] Kim W Egan JM The role of incretins in glucose homeostasis and diabetes treatment Pharmacol Rev 2008 60 470 512 10.1124/pr.108.000604 19074620 PMC2696340

[B13] Yamagishi S Matsui T Pleiotropic effects of glucagon-like peptide-1 (GLP-1)-based therapies on vascular complications in diabetes Curr Pharm Des 2011 17 4379 4385 10.2174/138161211798999456 22204436

[B14] Ikushima H Munakata Y Ishii T Iwata S Terashima M Tanaka H Schlossman SF Morimoto C Internalization of CD26 by mannose 6-phosphate/insulin-like growth factor II receptor contributes to T cell activation Proc Natl Acad Sci U S A 2000 97 8439 8444 10.1073/pnas.97.15.8439 10900005 PMC26966

[B15] Yamagishi S Nakamura K Matsui T Inagaki Y Takenaka K Jinnouchi Y Yoshida Y Matsuura T Narama I Motomiya Y Pigment epithelium-derived factor inhibits advanced glycation end product-induced retinal vascular hyperpermeability by blocking reactive oxygen species-mediated vascular endothelial growth factor expression J Biol Chem 2006 281 20213 20220 10.1074/jbc.M602110200 16707486

[B16] Sasaki N Takeuchi M Chowei H Kikuchi S Hayashi Y Nakano N Ikeda H Yamagishi S Kitamoto T Saito T Advanced glycation end products (AGE) and their receptor (RAGE) in the brain of patients with Creutzfeldt-Jakob disease with prion plaques Neurosci Lett 2002 326 117 120 10.1016/S0304-3940(02)00310-5 12057842

[B17] Tahara N Yamagishi S Takeuchi M Tahara A Kaifu K Ueda S Okuda S Imaizumi T Serum levels of advanced glycation end products (AGEs) are independently correlated with circulating levels of dipeptidyl peptidase-4 (DPP-4) in humans Clin Biochem 2013 46 300 303 10.1016/j.clinbiochem.2012.11.023 23219738

[B18] Ikushima H Munakata Y Iwata S Ohnuma K Kobayashi S Dang NH Morimoto C Soluble CD26/dipeptidyl peptidase IV enhances transendothelial migration via its interaction with mannose 6-phosphate/insulin-like growth factor II receptor Cell Immunol 2002 215 106 110 10.1016/S0008-8749(02)00010-2 12142042

[B19] Li H Zhang X Guan X Cui X Wang Y Chu H Cheng M Advanced glycation end products impair the migration, adhesion and secretion potentials of late endothelial progenitor cells Cardiovasc Diabetol 2012 11 46 10.1186/1475-2840-11-46 22545734 PMC3403843

[B20] Ueda S Yamagishi S Matsui T Noda Y Ueda S Jinnouchi Y Sasaki K Takeuchi M Imaizumi T Serum levels of advanced glycation end products (AGEs) are inversely associated with the number and migratory activity of circulating endothelial progenitor cells in apparently healthy subjects Cardiovasc Ther 2012 30 249 254 10.1111/j.1755-5922.2011.00264.x 21884000

[B21] Yan SF D'Agati V Schmidt AM Ramasamy R Receptor for advanced glycation endproducts (RAGE): a formidable force in the pathogenesis of the cardiovascular complications of diabetes &amp; aging Curr Mol Med 2007 7 699 710 10.2174/156652407783220732 18331228

[B22] Lin L Park S Lakatta EG RAGE signaling in inflammation and arterial aging Front Biosci (Landmark Ed) 2009 14 1403 1413 19273137 10.2741/3315 PMC2661616

[B23] Lamers D Famulla S Wronkowitz N Hartwig S Lehr S Ouwens DM Eckardt K Kaufman JM Ryden M Müller S Hanisch FG Ruige J Arner P Sell H Eckel J Dipeptidyl peptidase 4 is a novel adipokine potentially linking obesity to the metabolic syndrome Diabetes 2011 60 1917 1925 10.2337/db10-1707 21593202 PMC3121429

[B24] Ta NN Li Y Schuyler CA Lopes-Virella MF Huang Y DPP-4 (CD26) inhibitor alogliptin inhibits TLR4-mediated ERK activation and ERK-dependent MMP-1 expression by U937 histiocytes Atherosclerosis 2010 213 429 435 10.1016/j.atherosclerosis.2010.08.064 20843518

[B25] Shah Z Kampfrath T Deiuliis JA Zhong J Pineda C Ying Z Xu X Lu B Moffatt-Bruce S Durairaj R Sun Q Mihai G Maiseyeu A Rajagopalan S Long-term dipeptidyl-peptidase 4 inhibition reduces atherosclerosis and inflammation via effects on monocyte recruitment and chemotaxis Circulation 2011 124 2338 2349 10.1161/CIRCULATIONAHA.111.041418 22007077 PMC4224594

[B26] Stange T Kettmann U Holzhausen HJ Immunoelectron microscopic single and double labelling of aminopeptidase N (CD 13) and dipeptidyl peptidase IV (CD 26) Acta Histochem 1996 98 323 331 10.1016/S0065-1281(96)80025-0 8863861 PMC7131626

[B27] Ishibashi Y Matsui T Takeuchi M Yamagishi S Sitagliptin augments protective effects of GLP-1 against advanced glycation end product receptor axis in endothelial cells Horm Metab Res 2011 43 731 734 21932180 10.1055/s-0031-1284383

[B28] Yamagishi S Adachi H Nakamura K Matsui T Jinnouchi Y Takenaka K Takeuchi M Enomoto M Furuki K Hino A Positive association between serum levels of advanced glycation end products and the soluble form of receptor for advanced glycation end products in nondiabetic subjects Metabolism 2006 55 1227 1231 10.1016/j.metabol.2006.05.007 16919543

[B29] Nakamura K Yamagishi S Adachi H Matsui T Kurita-Nakamura Y Takeuchi M Inoue H Imaizumi T Serum levels of soluble form of receptor for advanced glycation end products (sRAGE) are positively associated with circulating AGEs and soluble form of VCAM-1 in patients with type 2 diabetes Microvasc Res 2008 76 52 56 10.1016/j.mvr.2007.09.004 18474381

[B30] Yamagishi S Matsui T Soluble form of a receptor for advanced glycation end products (sRAGE) as a biomarker Front Biosci (Elite Ed) 2010 2 1184 1195 20515790 10.2741/e178

[B31] Raucci A Cugusi S Antonelli A Barabino SM Monti L Bierhaus A Reiss K Saftig P Bianchi ME A soluble form of the receptor for advanced glycation endproducts (RAGE) is produced by proteolytic cleavage of the membrane-bound form by the sheddase a disintegrin and metalloprotease 10 (ADAM10) FASEB J 2008 22 3716 3727 10.1096/fj.08-109033 18603587

[B32] Iwaki-Egawa S Watanabe Y Kikuya Y Fujimoto Y Dipeptidyl peptidase IV from human serum: purification, characterization, and N-terminal amino acid sequence J Biochem 1998 124 428 433 10.1093/oxfordjournals.jbchem.a022130 9685737

[B33] Drucker DJ Nauck MA The incretin system: glucagon-like peptide-1 receptor agonists and dipeptidyl peptidase-4 inhibitors in type 2 diabetes Lancet 2006 368 1696 1705 10.1016/S0140-6736(06)69705-5 17098089

[B34] Baggio LL Drucker DJ Biology of incretins: GLP-1 and GIP Gastroenterology 2007 132 2131 2157 10.1053/j.gastro.2007.03.054 17498508

[B35] Ide Y Matsui T Ishibashi Y Takeuchi M Yamagishi S Pigment epithelium-derived factor inhibits advanced glycation end product-elicited mesangial cell damage by blocking NF-kappaB activation Microvasc Res 2010 80 227 232 10.1016/j.mvr.2010.03.015 20381502

[B36] Ojima A Ishibashi Y Matsui T Maeda S Nishino Y Takeuchi M Fukami K Yamagishi S Glucagon-like peptide-1 receptor agonist inhibits asymmetric dimethylarginine generation in the kidney of streptozotocin-induced diabetic rats by blocking advanced glycation end product-induced protein arginine methyltranferase-1 expression Am J Pathol 2013 182 132 141 10.1016/j.ajpath.2012.09.016 23159951

[B37] Yamagishi S Fujimori H Yonekura H Yamamoto Y Yamamoto H Advanced glycation endproducts inhibit prostacyclin production and induce plasminogen activator inhibitor-1 in human microvascular endothelial cells Diabetologia 1998 41 1435 1441 10.1007/s001250051089 9867210

[B38] Ishibashi Y Matsui T Takeuchi M Yamagishi S Glucagon-like peptide-1 (GLP-1) inhibits advanced glycation end product (AGE)-induced up-regulation of VCAM-1 mRNA levels in endothelial cells by suppressing AGE receptor (RAGE) expression Biochem Biophys Res Commun 2010 391 1405 1408 10.1016/j.bbrc.2009.12.075 20026306

[B39] Ojima A Matsui T Maeda S Takeuchi M Yamagishi S Glucose-dependent insulinotropic polypeptide (GIP) inhibits signaling pathways of advanced glycation end products (AGEs) in endothelial cells via its antioxidative properties Horm Metab Res 2012 44 501 505 22581648 10.1055/s-0032-1312595

[B40] Yamagishi S Amano S Inagaki Y Okamoto T Takeuchi M Makita Z Beraprost sodium, a prostaglandin I_2_ analogue, protects against advanced glycation endproducts-inducede injury in cultured retinal pericytes Mol Med 2002 8 546 550 12456993 PMC2040016

[B41] Kusunoki Y Hayashi T Morishita Y Yamaoka M Maki M Bean MA Kyoizumi S Hakoda M Kodama K T-cell responses to mitogens in atomic bomb survivors: a decreased capacity to produce interleukin 2 characterizes the T cells of heavily irradiated individuals Radiat Res 2001 155 81 88 10.1667/0033-7587(2001)155[0081:TCRTMI]2.0.CO;2 11121219

[B42] Lawson C Wolf S ICAM-1 signaling in endothelial cells Pharmacol Rep 2009 61 22 32 19307690 10.1016/s1734-1140(09)70004-0

[B43] Tuttolomondo A Di Raimondo D Pecoraro R Arnao V Pinto A Licata G Atherosclerosis as an inflammatory disease Curr Pharm Des 2012 18 4266 4288 10.2174/138161212802481237 22390643

[B44] Takenaka K Yamagishi S Matsui T Nakamura K Imaizumi T Role of advanced glycation end products (AGEs) in thrombogenic abnormalities in diabetes Curr Neurovasc Res 2006 3 73 77 10.2174/156720206775541804 16472128

[B45] Matsui T Nishino Y Takeuchi M Yamagishi S Vildagliptin blocks vascular injury in thoracic aorta of diabetic rats by suppressing advanced glycation end product-receptor axis Pharmacol Res 2011 63 383 388 10.1016/j.phrs.2011.02.003 21320599

[B46] Johansen OE Neubacher D von Eynatten M Patel S Woerle HJ Cardiovascular safety with linagliptin in patients with type 2 diabetes mellitus: a pre-specified, prospective, and adjudicated meta-analysis of a phase 3 programme Cardiovasc Diabetol 2012 11 3 10.1186/1475-2840-11-3 22234149 PMC3286367

[B47] Sakata K Hayakawa M Yano Y Tamaki N Yokota N Eto T Watanabe R Hirayama N Matsuo T Kuroki K Sagara S Mishima O Koga M Nagata N Nishino Y Kitamura K Kario K Takeuchi M Yamagishi SI Efficacy of alogliptin, a dipeptidyl peptidase-4 inhibitor, on glucose parameters, the activity of the advanced glycation end product - receptor for advanced glycation end product axis, and albuminuria in Japanese type 2 diabetes Diabetes Metab Res Rev 2013 10.1002/dmrr.2437 23861159

[B48] Raposeiras-Roubín S Rodiño-Janeiro BK Paradela-Dobarro B Grigorian-Shamagian L García-Acuña JM Aguiar-Souto P Jacquet-Hervet M Reino-Maceiras MV Alvarez E González-Juanatey JR Predictive value of advanced glycation end products for the development of post-infarction heart failure: a preliminary report Cardiovasc Diabetol 2012 11 102 10.1186/1475-2840-11-102 22909322 PMC3489693

[B49] Sarashina A Sesoko S Nakashima M Hayashi N Taniguchi A Horie Y Graefe-Mody EU Woerle HJ Dugi KA Linagliptin, a dipeptidyl peptidase-4 inhibitor in development for the treatment of type 2 diabetes mellitus: a phase I, randomized, double-blind, placebo-controlled trial of single and multiple escalating doses in healthy adult male Japanese subjects Clin Ther 2010 32 1188 1204 10.1016/j.clinthera.2010.06.004 20637971

